# Refractive Outcome of Phacoemulsification Cataract Surgery in Rural Sabah Using Immersion Biometry

**DOI:** 10.21315/mjms2021.28.5.9

**Published:** 2021-10-26

**Authors:** Jo Anne Sit, Sivaraj Raman, Arifah Nur Yahya

**Affiliations:** 1 Ophthalmology Department, Hospital Keningau, Keningau, Sabah, Malaysia; 2 Pharmacy Department, Hospital Keningau, Keningau, Sabah, Malaysia; 3 Ophthalmology Department, Hospital Queen Elizabeth, Kota Kinabalu, Sabah, Malaysia

**Keywords:** biometry, refractive error, cataract, rural, Sabah

## Abstract

**Background:**

A good refractive outcome after cataract surgery indicates adequate clinical service provision. Precise immersion biometry is critical to achieve the desired refractive outcome. While the immersion biometry results are good in the tertiary settings, it is of interest to explore the refractive outcome of cataract surgeries in a rural facility using the same technique.

**Methods:**

A retrospective cross-sectional review was conducted on medical records of all cataract surgeries carried out in Hospital Keningau, Sabah. This study used all patients’ medical records who had been assessed using immersion biometry pre-operatively, underwent phacoemulsification cataract surgery besides attending a post-operative refraction session within 90 days from the operation date. Clinical details were recorded in the form of standard proformas and analysed. The refractive outcome was evaluated using spherical equivalence (SE) and best-corrected visual acuity (BCVA). The percentage of cases with post-operative SE within ±1.00 diopter (D) and BCVA of ‘6/12 or better’ were determined. The association between demographic factors and surgical-related factors with post-operative SE was evaluated using Fisher’s exact test.

**Results:**

Of 140 cataract surgeries, 113 fulfilled the inclusion criteria. The average patient age was 66.3 (SD = 10.9) years old. The technique was proven to replicate a good outcome of 84.1% of cases with post-operative SE within ±1.00 D while 90.3% of the cases achieved BCVA of ‘6/12 or better*’*. Age and ethnicity were found to be associated with post-operative SE.

**Conclusion:**

The study proves the reproducibility of good refractive outcome in a rural facility using immersion biometry. The findings provide a benchmark for performance surveillance in rural facilities.

## Introduction

Based on the World Health Organization (WHO) (1) report in 2019, cataract was responsible for almost one-third of vision impairment worldwide, affecting about 65.2 million individuals. Even though it can be surgically removed, sociodemographic barriers often limit access to the treatment. This increases the burden of the disease in middle and low-income countries (2–3). The National Eye Survey (NES) II, for example, reported untreated cataracts as the leading cause of blindness in Malaysia (4). Consequently, many policies to increase cataract surgical coverage and rate are institutionalised in Malaysia and worldwide (5). While promoting cataract surgery as the primary focus to reduce blindness, the efforts to achieve the desired refractive outcome and visual acuity (VA) were also highlighted to ensure adequate clinical service provision in public hospitals (6).

Selection of the intraocular lens (IOL) power is one of the requisites to ensure accurate refractive outcome and good VA following cataract surgery (6). IOL power calculation is determined through pre-operative biometry that measures axial length and corneal radius of an eye. The data are then incorporated with the IOL power formula and A-constant to determine the final IOL power. Hence, the refractive outcome will be influenced by the accuracy of pre-operative biometry, which will in turn affect the final IOL power being selected.

Although the application of optical biometry for its superior consistency has been a global practice, immersion biometry has a better edge than optical biometry in measuring the axial length of dense cataracts (7–9). This is because the ultrasound produced during immersion biometry can transverse the dense cataract, but this is not the case for the laser beam produced during optical biometry (7). As those living in rural areas have a higher prevalence of mature cataracts than the urban population, immersion biometry remains relevant in such facilities (10). Additionally, immersion biometry is applied in many resource-limited settings as it requires lower equipment cost compared to optical biometry.

Refractive outcome has been applied as one of the metrics for appraising the service provision, as evidenced in the previous studies (6, 11–12). Gale et al. (11) and Murphy et al. (13) demonstrated the use of immersion biometry and non-customised A-constant for lens selection coupled with phacoemulsification to attain refractive outcomes of 72.3% and 80.3% cases with spherical equivalence (SE) within ±1.00 diopter (D) of target refraction respectively (11, 13). As for post-operative best-corrected visual acuity (BCVA), 86.9% of the cases achieved VA of ‘6/12 or better’ according to Murphy et al. (13). These studies were conducted at tertiary facilities with extensive resources, hence the reproducibility of these outcomes in a rural setting remains unknown. This reproducibility issue is important as rural facilities have constraints in terms of skilled manpower and accessible inventories. Therefore, it is of interest to explore the refractive outcome following phacoemulsification to establish a benchmark for the clinical service status in rural Malaysia.

## Methods

A retrospective, cross-sectional study was conducted at Hospital Keningau, a secondary referral hospital in Sabah. The eye care service in Hospital Keningau is provided by a team consisting of an ophthalmologist, an optometrist, medical officers as well as ophthalmic nurses. The arrangement of clinical personnel in the facility is in line with the model of secondary eye centre established by the International Centre for Advancement of Rural EyeCare (ICARE) (14). Phacoemulsifier machine, microscope and sterilisation units are available for cataract surgery setup. In addition, a slit lamp is utilised to evaluate ocular status pre-operatively and post-operatively.

The sample size was determined based on the ability to estimate 60% of patients undergoing cataract surgery via immersion biometry to achieve their target refraction. A lower proportion according to the finding from Gale et al. was applied to take into account the rural setting and sociodemographic characteristics (11). Based on the confidence level of 95% and the desired precision of 90%, the sample size was determined to be 93.

Medical records of the patients who had undergone cataract surgeries under the care of one optometrist and one surgeon from January to April 2018 were reviewed. The medical records were screened to include patients who had had immersion biometry, uneventful phacoemulsification, followed by a refraction session within 90 days of phacoemulsification.

Demographic data such as age, gender, ethnicity and pre-existing ocular comorbidity, pre-operative, intraoperative as well as post-operative details were recorded in a standard proforma. They were then analysed using SPSS Version 20.0 for Windows (IBM Corporation, New York, USA). Pre-operative data consist of corneal radius, axial length, A-constant, IOL formula, IOL model and IOL power selected by the surgeon. Intraoperative details such as complications and the IOL power being implanted were recorded. Post-operative details such as uncorrected visual acuity (UCVA), BCVA, subjective refraction and post-operative complications were followed up to determine the success of the treatment.

The Ophthalmology Department in Hospital Keningau manages in-house cataract surgeries for those living in the central rural zone of Sabah. Immersion A-scan and keratometer were the tools used to obtain the pre-operative biometry data in the setting. It was conducted using the immersion A-scan (Axis Nano, France) and Nidek KM-500 handheld automatic keratometer (Nidek, Japan). The inputs of A-constant and IOL formula were based on the reference provided by the IOL manufacturers and guidelines recommended by the Royal College of Ophthalmologists (RCOphth) (15). Any deviation from the reference was recorded as an error. Meanwhile, the IOL power implanted was compared to the IOL power chosen by the surgeon during the pre-operative assessment to determine any selection error. The surgical-related factors were analysed as binary variables and reported based on total percentage.

**Figure 1:**
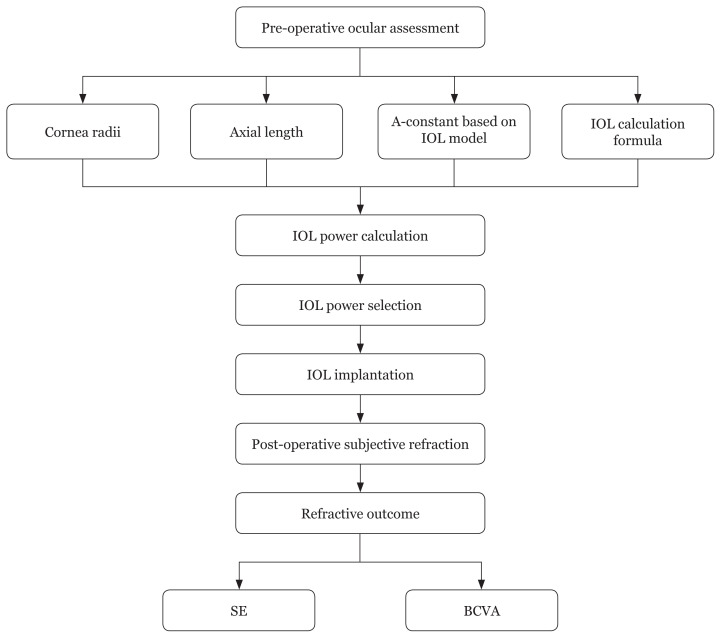
Flow chart of refractive outcome assessment

Refractive outcome was evaluated using SE and BCVA. The cases with post-operative SE that fell within ±1.00 D of target refraction were identified. Also, the percentage of BCVA of ‘6/12 or better’ was recorded. The data were evaluated as dichotomous variables. The thresholds of ‘±1.00 D’ and ‘6/12’ were set to allow for a standard comparison with other studies (11, 13). The flow chart of the refractive outcome assessment is shown in Figure 1. The association between demographic factors and surgical-related factors with post-operative SE was evaluated using Fisher’s exact test. A *P*-value of less than 0.05 was set for statistical significance.

## Results

Of 140 cataract cases, 113 fulfilled the inclusion criteria. The average age of patients at the time of surgery was 66.3 (SD = 10.9) years. Gender was almost equally distributed, while in terms of ethnicity, the majority were of Dusun lineage (34.5%), followed by Murut (31.9%), Chinese (15.9%) and others (17.7%).

Pre-operatively, 11 cases (9.7%) had pre-existing ocular diseases such as advanced glaucoma, corneal scars, chronic anterior uveitis, macular hole and wet age-related macular degeneration. There were 12 cases (10.6%) of A-constant input error during IOL power calculation, five cases (4.4%) of IOL calculation formula selection error while no error was made for corneal radius. An error (0.9%) of non-matching IOL power implantation was observed but there was no intraoperative complication. Four foldable monofocal IOL models (Tecnis ZCB00, Akreous Adapt AO, Aurovue and ORIZON) were used for all cases of cataract surgeries in the rural facility.

**Figure 2:**
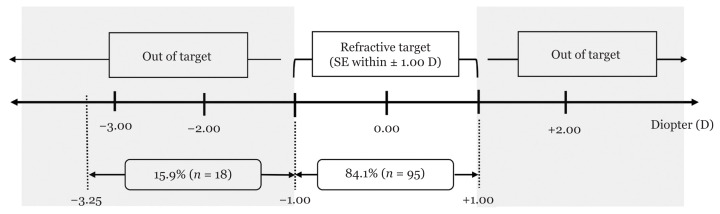
Percentage of cases with post-operative SE that fell within ±1.00 D of target refraction

**Figure 3:**
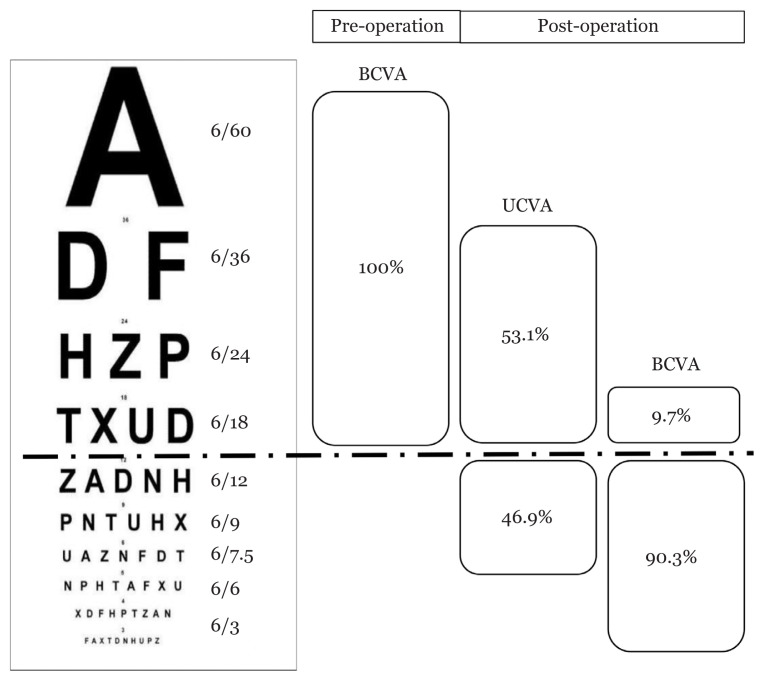
Distribution of VA before and after cataract operation. The threshold was based on the standard of achieving a VA of ‘6/12 or better’

The percentage of post-operative SE that fell within ±1.00 D is shown in Figure 2. The average difference between the pre-operative target refraction and actual post-operative refraction was −0.47 D (SD = 0.75), ranging from −3.25 D to +1.00 D. Figure 3 depicts the distribution of the VA before and after phacoemulsification. Forty-nine eyes (43.4%) required glasses to achieve VA of ‘6/12 or better*’*. On the other hand, 11 eyes (9.7%) with pre-existing ocular diseases failed to achieve the VA threshold even with glasses.

In this study, age and ethnicity were found to be associated with post-operative SE. Patients younger than 65 years old tend to achieve SE within ±1.00 D after cataract surgery. Likewise, Murut patients had the highest cases (97.2%) to achieve the targeted refractive outcomes, followed by Chinese (83.3%) and Dusun (71.8%). The association between demographic characteristics and the surgical-related factors with post-operative SE is highlighted in Table 1.

## Discussion

Many patients expect to be glasses-free with their sight restored upon successful completion of cataract surgeries. Hence, achieving target refraction via the correct selection of IOL power reflects the quality of clinical service. In this study, it was shown that immersion biometry and non-customised A-constant for lens selection coupled with phacoemulsification in the rural facility were able to achieve 84.1% post-operative SE within ±1.00 D of target refraction. This was higher than the results reported by Gale et al. (11) and Murphy et al. (13), which were 72.3% and 80.3%, respectively (11, 13). Both studies were set as the benchmark for performances, as they were conducted by tertiary teaching hospitals in England.

It is postulated that the better performance in this study was due to the positive relationship between case volume and outcome. The more a surgeon performed the procedure, the greater experience gained was translated into improved outcome (16). In this facility, the ratio of a surgeon to cataract surgeries conducted in 2018 was 1 to 246 cases. Moreover, all cataract assessments and surgeries were carried out by a single optometrist and surgeon. Such practice reduced the operator variability. In other words, multiple surgeons and optometrists may lead to higher variation, thus reducing the consistency as reported in other studies.

Meanwhile, only 46.9% of patients achieved post-operative UCVA of ‘6/12 or better’. This was lower than the 56.3% reported by Murphy et al. (13). Patients’ age and pre-existing corneal astigmatism were found to be potential contributors to the reduced UCVA in several studies (3, 17–18). Older patients especially those above the age of 65 years old have significantly poorer UCVA (3, 18). Furthermore, patients with pre-existing corneal astigmatism have perceived blurry vision post-operatively as little as 0.75 D astigmatism (17). Further study is required to identify factors that contribute towards the reduced UCVA in Hospital Keningau so that remedial measures can be taken. Nevertheless, 90.3% of patients achieved BCVA of ‘6/12 or better’, in line with the threshold used by Murphy et al., in which 86.9% of the patients achieved BCVA of ‘6/12 or better’ (13).

Interestingly, the refractive outcome in this study was skewed towards myopia. The incidences of post-operative myopic shift could be due to the application of non-customised A-constant (6). Non-customised A-constant hardly yields an optimal refractive outcome due to variation in measurement and surgical techniques by optometrists and surgeons respectively (19). Thus, it is recommended to rectify such myopic shift via customisation of A-constant based on the previous refractive outcome.

On top of that, adjustment of the first eye prediction error by 50% during the IOL selection for the second eye was suggested to further enhance the refractive outcome (6, 20–21). In fact, if the refractive outcome of the first eye deviates by −1.00 D from the initial target, the second eye should be aimed at least +0.50 D from the target refraction. The partial adjustment can compensate for the unexpected prediction errors made during pre-operative biometry measurement and uncontrolled individual ocular conditions (20). The individual ocular anatomy will cause variation in the post-operative position of IOL in the eye and thus, deviates the refractive outcome (21). Given that the anatomy of an eye resembles its pair in an individual, the partial compensation for the target refraction of the second eye based on the first eye is useful in minimising the refractive deviation.

The study also found that age and ethnicity were associated with the refractive outcome. A previous study by Simon et al. (12) showed that older age was associated with worse surgical outcomes. This was contributed by poor cooperation during pre-operative biometry, especially immersion biometry as the contact technique that demands higher cooperation from patients. In addition to age, the refractive outcome was significantly influenced by ethnicity, which was corroborated by Thevi and Godinho (22). A majority of Murut patients achieved target refraction compared to other races. However, the underlying cause of this finding is yet to be identified nor explained in the literature. The significance could have been attributed to the socioeconomic status and lifestyles, which were not explored in the study. Further studies with the application of robust statistical analysis such as multiple linear regression are required to determine relationships and interactions, especially between ethnicity and co-factors.

This study also detected inaccuracies in A-constant input, IOL formula and IOL implanted. However, these errors did not affect the refractive outcome significantly. Even though the preliminary findings did not show the association between the errors and refractive outcome, it highlighted the possible area for improvement. Pre-operative biometry countercheck protocol can be developed in rural facilities to prevent such lapses from happening.

There were several limitations stemming from this retrospective observational study design. As the data were obtained from the past cases, there was a lack of control on patient characteristics where the heterogeneity level of patients beyond the factors explored was uncertain. Furthermore, the sample size may not be powered to investigate the differences in sociodemographic and surgical factors. As most institutions in Malaysia adopt and apply only a single biometry technique in service to ensure consistency, future cohort-based studies conducted at multiple centers are recommended to further compare and contrast these findings.

In conclusion, this study has proven the reproducibility of good refractive outcomes in a rural facility using immersion biometry technique. Our findings were shown to be comparable and even better than the previous studies applying a similar standard of practice. Thus, the reported values can be used as a benchmark or target for performance surveillance in facilities sharing the similar setting and framework. The highlighted limitations and operative errors also contribute towards a larger pool of knowledge on cataract surgeries in rural facilities while identifying areas for remedial actions.

## Figures and Tables

**Table 1:** Association between demographic and cataract surgery-related factors with post-operative SE

Variable		*n*	Post-operative SE		

			Within ± 1.00 D *n* (%)	Out of ± 1.00 D *n* (%)	*P*-value
Age	< 65	51	47 (92.2)	4 (7.8)	0.040^a^
	≥ 65	62	48 (77.4)	14 (22.6)	
Gender	Male	55	48 (87.3)	7 (12.7)	0.445
	Female	58	47 (81.0)	11 (19.0)	
Ethnicity	Dusun	39	28 (71.8)	11 (28.2)	0.017^a^
	Murut	36	35 (97.2)	1 (2.8)	
	Chinese	18	15 (83.3)	3 (16.7)	
	Others	20	17 (85.0)	3 (15.0)	
Pre-existing ocular comorbidity	No	54	43 (79.6)	11 (20.4)	0.224
	Yes	28	23 (82.1)	5 (17.9)	
	No fundus view	31	29 (93.5)	2 (6.5)	
A constant	Correct	101	84 (83.2)	17 (16.8)	0.687
	Error	12	11 (91.7)	1 (8.3)	
IOL formula	Correct	108	90 (83.3)	18 (16.7)	1.000
	Error	5	5 (100)	0 (0.0)	
IOL implanted	Correct	112	95 (84.8)	17 (15.2)	0.159
	Error	1	0 (0.0)	1 (100)	

Note:

a statistically significant association based on Fisher’s exact test (*P* ≤ 0.05)
